# Evaluating Pre-travel Health Consultations for Business and Occupational Travelers: A Systematic Review

**DOI:** 10.7759/cureus.70991

**Published:** 2024-10-07

**Authors:** Cyprel Ijeh, Damayantha Yohan Watthuhewa

**Affiliations:** 1 Pharmaceutical Medicine, Stemax Consult Healthcare Services Ltd, Milton Keynes, GBR; 2 Obstetrics and Gynaecology, George Eliot Hospital, Nuneaton, GBR

**Keywords:** business travelers, occupational travelers, pre-travel consultation, risk assessment, risk management

## Abstract

Business and occupational travelers’ health is at risk due to the specific itineraries and activities, prolonged stays, work-related stressors, short preparation time, more chances of disease importation, underutilization of vaccination, and chemoprophylaxis. The objective of the review is to assess the effectiveness of pre-travel health consultation and how it will help travelers prevent health risks. The question is to evaluate how can prolonged stays and underutilization of chemoprophylaxis and vaccination be better managed with pre-travel health consultation.

The literature was searched on databases such as PubMed, Google Scholar, Cochrane Library, and Semantic Scholar using Boolean operators with keywords and Medical Subheading (MeSH) terms such as "occupational travelers," "business travelers," "pre-travel health consultation," "effectiveness of consultation," "health risk assessment," "travel illness prevention," "risk management," and "risk assessment" to retrieve relevant published studies. The Cochrane Risk of Bias (ROB) 2.0 tool and Newcastle Ottawa scale (NOS) were utilized to measure the risk of bias. The Grading, Recommendation, Assessment, Development, and Evaluation (GRADE) tool was used to assign evidence strength. In total, a preliminary search yielded 334 articles. One high-quality study and seven studies of moderate quality were included.

In conclusion, pre-travel health consultations are a vital tool to prevent travel-related health problems in business and occupational travelers. The current approach needs to be more specific and proactive to address health-specific risks experienced by travelers. However, early comprehensive consultations focusing on preventive measures, region-specific health risks, and timely immunizations are crucial to improving health outcomes. Moreover, enhanced guidance, awareness, and education of health professionals are also necessary to treat the complex medical needs of business and occupational travelers effectively.

## Introduction and background

Pre-travel health consultations are essential for business and occupational travelers to reduce health risks [1, 2]. These consultations include risk assessments, preventive measures such as immunization recommendations and chemoprophylaxis, safe and healthy travel experiences, productivity, and the well-being of travelers [3]. However, the literature indicates that only 45% of business travelers seek pre-travel healthcare interactions, which results in underutilizing preventive interventions [4]. Interventions like text message reminders have been shown to increase attendance at sexual health consultations, implying that similar tactics may be used to promote pre-travel health consultations [5]. Furthermore, age and previous travel history affect the chances of seeking pre-trip health consultations. Therefore, raising awareness of health hazards is critical for effective risk communication to ensure the overall success of business operations and productivity. It may enhance well-being during pre-travel consultations [6].

The overall scope of pre-travel health consultation is not limited to infectious diseases but also ranges beyond them. Although infectious diseases are the primary focus on most occasions, there is also a need to recognize and highlight the broader spectrum of health concerns that may impact travelers' health. Therefore, there is a need to highlight the scope of pre-travel health issues beyond infectious diseases. It may also focus on injuries and accidents (slips, falls, transportation-related accidents in specific work and environment) [7], non-communicable diseases (cardiovascular diseases, diabetes, respiratory disorders) [8], environmental-related hazards (sudden variability in temperature, altitude sickness, air pollution, natural disasters) [9], psychological well-being (tight schedules, unfamiliar surroundings, work-related pressures), and health insurance related to travel [10]. Hence, the comprehensive evaluation of these factors by addressing diverse health concerns and infectious diseases may improve the health and overall well-being of business and occupational travelers. Pre-travel health consultation is necessary to provide complete insights to address challenges for travelers. It must underscore awareness regarding possible health risks, optimized management of non-communicable diseases, strategies to mitigate risks with the management of potential health consequences, and informed about the range of insurance coverage to meet emergencies, medical expenses, and repatriation in case of illness on foreign territory [10]. 

The rationale behind this review is to assess pre-travel health consultation effectiveness and how it may help business and occupational travelers to prevent travel-related illnesses, infections, and factors that could cause health risks among them. Occupational and business travelers often face significant health risks due to specific itineraries and activities. It necessitates tailored pre-travel health consultation for them to mitigate risks effectively due to their prolonged stays, work-related stressors (visiting more endemic areas due to occupation or business requirements), shorter preparation time, and potential for the importation of disease into communities as compared to tourists and students [11]. Moreover, Chen et al. (2018) also highlighted that the adoption of preventive measures, e.g., vaccinations and chemoprophylaxis, is underutilized among business travelers, which exposes their health to more risk [3].

Objective

This systematic review aims to assess how pre-travel health consultation helps business and occupation travelers prevent health risks. The review assesses how prolonged stays and underutilization of chemoprophylaxis and vaccination can be better managed with pre-travel health consultation and the limitations and challenges of providing pre-travel health consultations for occupational and business travelers.

## Review

The systematic review was conducted using Preferred Reporting Items for Systematic Review and Meta-analysis (PRISMA) guidelines, 2020 [12].

Search strategy 

The literature was searched on databases such as PubMed, Google Scholar, Cochrane Library, and Semantic Scholar using Boolean operators with keywords and Medical Subheading (MeSH) terms to retrieve relevant published studies. Open-access, full-text available English-language articles on humans from 1 January 2014 to 30 May 2024 limiters were applied to the database search. To formulate research questions that were specific and answerable, the Patient/Problem/Population, Intervention/Exposure, Comparison, Outcome (PICO) framework was used to structure and refine the question. The PICO criteria are as follows: Population/problem: occupational and business travelers; Intervention: pre-travel health consultation; Comparison: standard advice/none; Outcomes: health risk assessment, the effectiveness of consultation, and illness prevention.

Relevant keywords, text words, and controlled vocabulary used to retrieve literature are mentioned in Table 1.

**Table 1:** Keywords utilized for relevant literature search “-” showed empty cell with no MeSH term or controlled vocabulary retrieved MeSH: Medical Subheading

Concept	Text words	Controlled vocabulary (MeSH terms)
Problem/population	Occupational, business, and corporate travelers	-
Intervention	Pre-travel consultation, pre-travel advice, pre-travel preparation	“Travel medicine”, "Referral & Consultation"
Comparison	Standardized advice/none	Standardized advice/None
Outcomes	“Risk assessment”: risk perceptions assessment of healthcare	“Health occupations”, “Travel-related illness”, "Risk assessment", “Prevention and Control (subheading)”

Formulation of the research question

How does pre-travel health consultation help business and occupation travelers prevent health risks? What are the limitations and challenges of pre-travel health consultations for business and occupational travelers?

Inclusion criteria

Randomized controlled trials (RCTs), prospective cohort studies, retrospective observational publications, and surveys relevant to the review objective were included. Studies focused on business and occupational travelers were included. Studies in which pre-travel health consultation, including assessments of vaccinations, preventive measures, and health advice given before travel, were included. Studies that assess the comprehensiveness and quality of pre-travel health consultations, including assessment of consultation-related content, adherence to practice guidelines, patient satisfaction, and health outcomes, were also included. Open-access articles with full text available in the English language on humans from 2014 to 2024 were included.

Exclusion criteria

Case reports, case series, and review articles were excluded. Studies were conducted on populations other than occupational and business/corporate travelers, such as students, immigrants, pilgrims, and tourists. Studies that are paid were not included because of their inaccessibility to complete data findings and limited resources. Studies with unclear and insufficient methodology data were also excluded. Studies written in languages other than English were excluded.

Studies selection process

The PRISMA Guidelines 2020 were followed for literature identification, screening, selection, and reporting [12]. After identifying relevant literature, only studies that met inclusion criteria during screening were included. Then, selected studies underwent quality assessment. The selection process involved two independent reviewers.

Quality assessment

The quality assessment was carried out by two independent reviewers. The Cochrane Risk of Bias (ROB) 2.0 tool was also used to determine ROB among RCTs using five domains [13]. Moreover, the Newcastle-Ottawa Scale (NOS) was used to evaluate the non-randomized studies, such as cohort and cross-sectional studies. The two independent reviewers evaluated three domains of NOS: selection (a maximum score of four), comparability (a maximum score of two), and outcomes (a maximum score of three). A score of 0 to four indicates a high risk of bias, five to six indicates some concern about the risk of bias, and a score of seven to nine indicates a low risk of bias [14]. The non-randomized controlled trial quality was assessed using the Cochrane ROB 2.0 tool using five domains to determine the risk of bias [15]. The Grading of Recommendations Assessment Development and Evaluation (GRADE) tool was used to determine evidence strength. The GRADE tool rated the strength of evidence recommendation on the basis of five domains: risk of bias, inconsistencies, indirectness, imprecision, and publication bias. The GRADE tool rated studies evidence into four categories: high, moderate, low, and very low, via assessing these five domains [16]. 

Data extraction and synthesis

Thematic analysis, an inductive, data-driven strategy was used for analysis by two independent reviewers [17]. There was no disagreement reported among the two reviewers, and any conflict was resolved through double-checking and consensus. Quantitative data were extracted and entered into a spreadsheet, including information related to study design, characteristics, sample size, intervention characteristics, and the results of the studies. The analysis consisted of a step-wise approach. It includes familiarization with the data, repeated review of the study’s findings, analyzing, and reviewing themes via an iterative process. The analysis of key themes was done to examine the findings of the studies critically. The themes that contributed to answering the pre-defined research question or focusing on the objective of the systematic review were analyzed comprehensively to critique the results. It helped synthesize evidence through a comprehensive narrative approach surrounded by a critical perspective to ensure evidence-based practice. 

Ethical consideration

**Figure 1:**
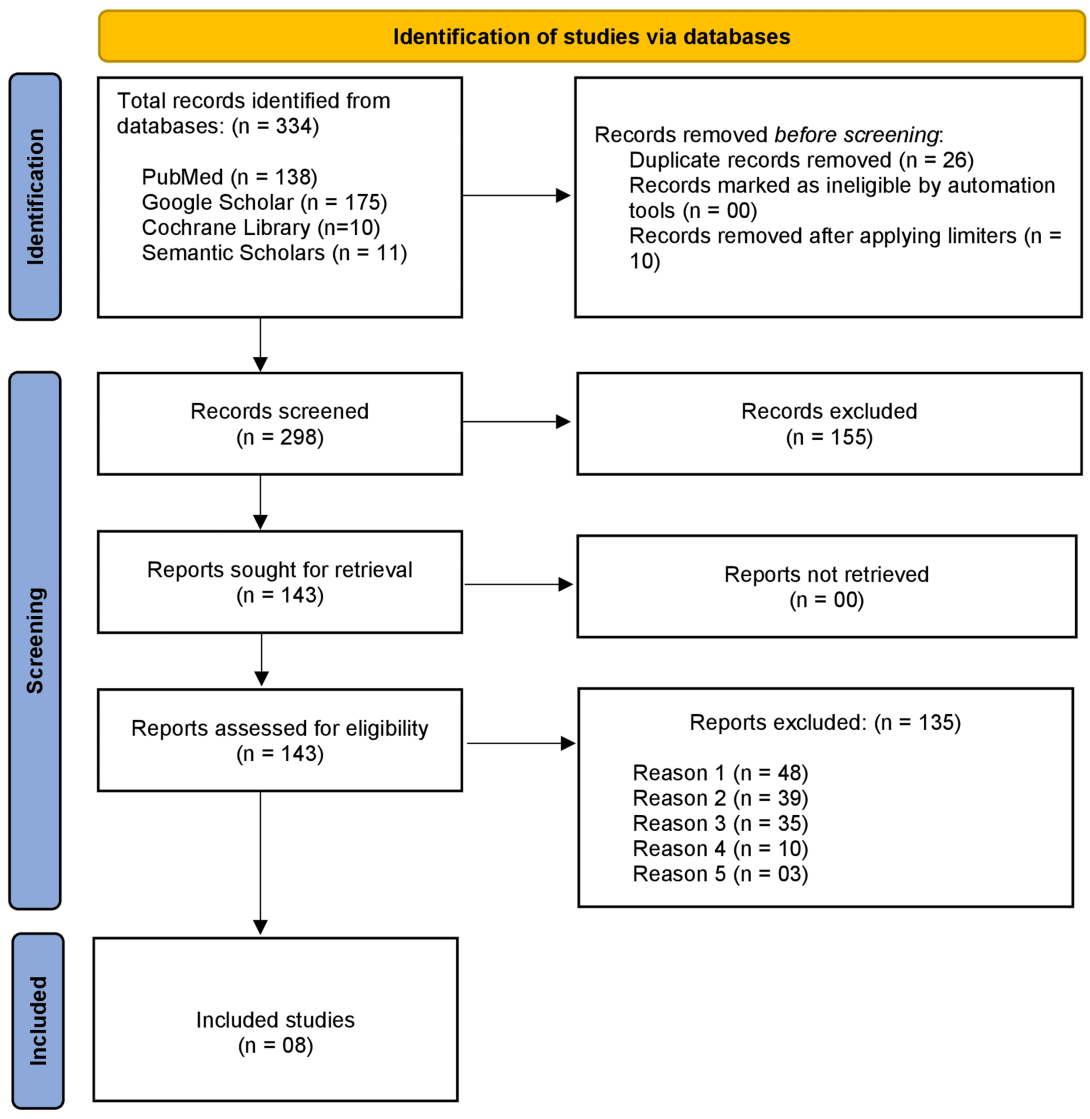
The PRISMA flowchart outlining the study selection process Reason 1: study designs (case reports, case series, reviews, editorials, and comments); Reason 2: irrelevant outcomes; Reason 3: studies related to tourists, students, no-pre-travel health consultation, studies of non-travel-related illnesses; Reason 4: low-quality studies; Reason 5: very low-quality studies PRISMA: Preferred Reporting Items for Systematic Review and Meta-analysis

The participants were not directly involved in gathering data to write this review. The data were extracted from already published literature. However, research on human-based interventions was taken into account. Determining the baseline homogeneity is another important step in assessing the genuine post-intervention/exposure effect. Moreover, data about the privacy and confidentiality of participants would be concealed to ensure anonymity. Lastly, the authors have not disclosed any conflicts of interest during the selection and quality assessment of included studies following PRISMA 2020 guidelines mentioned in Figure 1.

Results

Three hundred and thirty-four articles were retrieved on initial search using keywords and MeSH terms on Google Scholar, PubMed, Cochrane Library, and Semantic Scholar. Twenty-six duplicates and 10 articles were eliminated after applying limiters and duplication tools. The next step was to screen 298 articles. One hundred and fifty-five articles were eliminated due to non-relevance. The eligibility of the remaining 143 articles was determined. One hundred and thirty-five articles were excluded because they did not qualify for the inclusion criteria. Furthermore, 21 studies were chosen for quality assessment. The NOS and Cochrane ROB 2.0 tool were used for risk of bias assessment. The GRADE tool categorized the articles into one high-quality, seven moderate-quality, 10 low-quality, and three very low-quality studies. Finally, one high-quality study and seven moderate-quality studies were included.

Characteristics of the included studies

The review involved eight studies. One study was a randomized controlled trial, and seven were non-randomized controlled trials. A total of 24,456 participants were considered in this review from seven different geographical regions. The characteristics of the included studies are mentioned in Table 2.

**Table 2:** Characteristics of the included studies NOS: Newcastle-Ottawa Scale; ROB: Risk of Bias; GRADE: Grading of Recommendations Assessment Development and Evaluation; M: male; F: female; H1N1: swine flu

Serial #	Author, year study	Country of origin	Study design	NOS scale	Cochrane ROB 2.0 tool	Evidence strength (GRADE)	Sample characteristics and gender (M/F)	Objectives	Limitations
1	Shady et al. (2015) [18]	Kuwait	Analytical comparative study	5	-	Moderate quality	800 people	Predictors for receiving a pre-travel consultation	Selection bias, the H1N1 pandemic limits traveling, limited morning working hours
2	Weitzel (2020) [19]	Chile	Cross-sectional study	6	-	Moderate quality	1,341 travelers; M: 657, F: 684	Pre-travel advice complexity, vaccination needs	Single-center with one travel medicine expert involved
3	Rozenberg et al. (2019) [20]	Israel	Randomized controlled trails	-	Some concerns	High quality	75 participants	Role of online consultation	Limited face-to-face interaction, incomplete recall in travelers being unsure, higher drop-out rates, non-validated interviews, and small sample size
4	Tan et al. (2018) [21]	USA	Retrospective cohort study	6	-	Moderate quality	1,160 participants	Pre-travel consultation's impact on clinical outcomes and management of diarrhea among travelers	Missing participants data, lacking healthy control groups, unable to determine the true burden of disease.
5	Vilkman et al. (2016) [22]	Finland	Prospective study	5	-	Moderate quality	460 subjects	Risk factors and advice for pre-travel	Overrepresented visitors, small sample size, limited follow-up period
6	Han et al. (2015) [23]	Ireland	Cross-sectional observational	5	-	Moderate Quality	4,817participants records	To characterize preexisting medical conditions and medications of travelers seeking pre-travel health advice	Cross-sectional study design, inherent bias due to retrospective study design
7	Shaw et al. (2015) [24]	New Zealand	Retrospective observational study	6	-	Moderate quality	363 individuals	To pre-travel awareness travelers about the prevention of the spread of diseases	Inherent information bias due to study design
8	Walker et al. (2015) [25]	USA	Multicentre cross-sectional observational study	6	-	Moderate quality	15,440 travelers	To analyze vaccines, travelers focus on the influence of time to departure and travel purposes	Lacked detailed descriptions regarding itinerary, accommodations, and activities. Retrospective design inherent information bias, limited data exist on the safety and vaccine interchangeability, Japanese encephalitis, and rabies

The evidence is synthesized into thematic areas that provide complete insights into the impact of pre-travel health consultation on business and occupational travelers. It also illuminates the relevance of pre-travel health consultation to improve health outcomes. Each theme has been presented in a separate paragraph to demonstrate its role (Table 3).

**Table 3:** Main findings and sub-themes of the included studies

Serial #	Author, year study	Sub-themes	Main findings
1	Shady et al. (2015) [18]	Demographic and travel factors as predictors of pre-travel health consultation	Purpose of traveling, nationality, time of stay, and destination spot of travel are all statistically significant with pre-travel health consultation (p<0.05). These are all predictors of assessing travel risk behaviors for assessing travel health consultation (p<0.05).
2	Weitzel (2020) [19]	Pre-travel health consultation complexity	Over 60% of consultations were regarded as complex to assess the profile and complexity of pre-travel consultations. In the analysis, the majority (90%) were adults, with the most (80.3%) being aged 18-59 years and 6.3% aged 60-69 years.
3	Rozenberg et al. (2019) [20]	Awareness and knowledge about travel hazards	The standard consultation control group had better awareness of travel-related hazards before traveling (P = 0.014). There was a trend towards higher levels of knowledge about travel risks throughout the trip (P = 0.06).
4	Tan et al. (2018) [21]	Improvement of health outcomes	Stool tests and antibiotic prescriptions increased with pre-travel consultation (odds ratio (OR) 1.6 (95% CI 1.1–2.4), (OR 1.6 (95% CI 1.1–2.5). The pre-travel consultation group had shorter hospital stays (mean 1.8 days vs. 3.3 days for non-pre-travel, p = 0.006) and lower gastrointestinal consultation rates (OR 0.4 (95% CI 0.2–0.9)).
5	Vilkman et al. (2016) [22]	Persistent illness and predisposing factors	Despite pre-travel consultations and vaccine prophylaxis, there is still illness presented among most of the travelers when examining their behavior and illness symptoms. The predisposing factors responsible for illness were specific regions (southern, east-southern Asia, eastern Africa), females, young age travelers, and length of stay. The longer the stay, the 2.5% chance of contracting an illness increased each day. Age group and travel duration were statistically significant (p<0.05).
6	Han et al. (2015) [23]	Pre-travel medical health and education of travel health professionals	This study reveals travelers’ medical profiles at travel clinics. The variety of ailments described emphasizes the need to educate doctors and nurses about travel health concerns for specific conditions. Pre-trip health consultations will consider how travel affects medical issues.
7	Shaw et al. (2015) [24]	Inadequate vaccination and care after exposure	Travelers need improved guidance on travel-related rabies vaccination since WHO guidelines are not followed abroad. Few travelers obtained pre-travel immunization, and just 20.3% received WHO-advised postexposure care. Thus, 79.7% of the cohort were potentially at risk for rabies due to improper care after exposure.
8	Walker et al. (2015) [25]	Effectiveness and timing of pre-travel health consultation	Due to time constraints and low vaccination rates, travelers should be aware of earlier pre-travel consultations at least four to six weeks before the trip and precise risk assessment. Effective vector avoidance, rabies, and animal bite prevention and management counseling is essential.

Shady et al. (2015) found that nationality, purpose of the trip, duration of stay, and destination are key factors in whether travelers seek pre-travel health consultations. Business and occupational travelers were at risk because of their specific needs of travel requirements that emphasize the use of pre-travel health consultation. These parameters can help to predict the health risks during travel, which necessitates the targeted pre-travel health consultation [18].

Weitzel (2020) found more than 60% of pre-travel health consultations were complex due to different health profiles among travelers. These travelers, occupational and business, often engage in risky activities and visit endemic areas that present the need for complex health needs. Therefore, there is a need to tailor pre-travel health consultations either through campaigning or awareness programs to address the complexity of the itinerary as well as the health profile of travelers to ensure adequate preventive measures, e.g., vaccination and prophylaxis [19]. However, these facilities are limited in less developed regions which pose a challenge. A comprehensive strategy is needed to prevent the spread of health diseases to address the complexity of things such as records, interpretation of records, and making of policies as per records.

Rozenberg et al. (2019) highlighted the importance of awareness and knowledge of travel-related hazards through pre-travel health consultation. Its application showed better awareness of travel-related illness before and during trips. Pre-travel health consultation is particularly relevant for business and occupational travelers who lack health risk awareness due to a short or limited time of preparation. It will provide an essential awareness program to inform them about region-specific diseases and preventive measures to mitigate health risks that may be encountered while on work [20].

Tan et al. (2018) added further through findings of the research that the business and occupational travelers who attend pre-travel health consultation experienced better health outcomes, such as shorter hospital stays and a lower incidence of gastrointestinal problems during and after returning from trips, as compared to those who did not attend pre-travel health consultation. The better health outcomes also reduced work disruptions, medical emergencies, and the cost of health care while traveling. Therefore, it is a supported argument that awareness and knowledge training by pre-travel health consultation not only prevent illness but also ensure safer travel experiences, ultimately improving health outcomes [21].

However, Vilkman et al. (2016) also presented other aspects, as business and occupational travelers still contracted an illness while on travel despite receiving pre-travel health consultations and vaccinations. This finding suggests there is a need to evaluate the pre-travel health consultation program to provide more specific advice and preventive measures due to prolonged stays and more frequent visits to endemic regions. Therefore, the findings highlight that pre-travel health consultation may not always provide 100% safety, and there is a need for additional strategies to prevent illness [22]. Moreover, the authors also highlighted that the ineffectiveness of pre-travel health consultation is also due to limited government policies and national guidelines, especially in developing nations, that are beyond the control of travelers.

Han et al. (2015) also highlighted that occupational and business travelers often reported pre-existing medical health conditions while attending pre-travel health consultations. It may be one of the reasons that they suffer more health issues during or after travel despite pre-travel consultation. It points out the need for a comprehensive consultation to address how their pre-existing health issues may interact with travel-related risks. However, the authors also found that most healthcare professionals are also not appropriately educated on treating or handling complex cases, especially these work travelers. Therefore, there is a need to educate healthcare professionals who can guide them adequately and provide solutions [23].

Shaw et al. (2015) also demonstrated some issues with not receiving pre-travel vaccination and post-travel management. It will leave their health at risk. The authors highlighted that occupational and business travelers visiting regions are exposed to rabies due to interaction with animals in certain industries. The one main reason is also highlighted: only one-fifth of the travelers get post-exposure management after returning from a health-risk area, putting them at risk of health travel-related issues. Therefore, there is a need to implement policies to enforce post-exposure care because it may also put other individuals’ health at risk [24].

Walker et al. (2015) presented a solution to address the limited time of preparation among travelers. The authors stressed the importance of scheduling pre-travel health consultations before four to six weeks of departure because most of the work or business travels are pre-scheduled. It will help these travelers to make timely consultations, which is critical for adequate risk assessment and immunization. It will underscore the prompt need for accessible consultations that will be tailored to the compressed schedules of business and occupational travelers [25].

Discussion

The synthesis of evidence, including eight studies, suggests that pre-travel health consultations are as effective in improving awareness as improving health outcomes. The demographic and travel-related predictors are nationality, travel purpose, stay length, location, and individualized healthcare services. However, there are gaps indicated by the evidence to tailor pre-travel consultations as per the specific needs of business and occupational travelers. Unique travel patterns, prolonged stays in high-risk regions, and compression preparation time need a more structured as well as proactive approach to pre-travel health consultations. Vaccination remains low, preventive measures are also underutilized, and pre-travel health consultations are often taken too late to be fully effective. The business and occupational travelers complexity of travel needs and pre-existing health profiles demands a more comprehensive and tailored approach to pre-travel health consultation.

In a study by Shady et al. (2015), education about health and destination spots is significantly associated with the tendency of travelers to visit Travel Health Clinics (THCs) [18]. A similar association was also reported by Toovey et al. (2004) [26]. Shady et al. (2015) found no significant impact of age factor among travelers, despite having active physical health and stable financial sources, on seeking pre-travel health consultation (p > 0.05). These findings are aligned with the studies carried out in Quebec [27], Singapore [28], and Germany [29], which reported that awareness and knowledge of health risks are the factors to advise travelers to seek pre-travel health consultation.

Shady et al. (2015) emphasized that the implementation of preventive measures can be associated with perceptions of travelers, risks, and challenges specific to the destination. Similarly, the World Health Organization (WHO) emphasizes preventing infections such as meningococcal meningitis, yellow fever, and malaria by providing prophylaxis for specific destinations [30, 31]. Therefore, travelers must be aware of and know the risks to their health to engage them to seek travel health consultation. Gesser-Edelsburg et al. (2014) also highlight similar practices for tourists to obtain typhoid immunizations, hepatitis A vaccines, and diphtheria and tetanus (D-T) toxoids during the swine flu (H1N1) influenza pandemic [30]. Therefore, the strategies to implement preventive efforts must consider all discussed aspects to ensure healthcare among travelers [19]. The preventive efforts can be done more comprehensively through the engagement of travelers by providing knowledge and guiding them regarding post-exposure management. Lastly, Shady et al. (2015) also recognize specific drawbacks in their study. This study was undertaken at the onset of the H1N1 flu pandemic in Mexico and America, which had a substantial influence on travel. The impact was seen through a decline in the volume of visitors to the THC. The study was observational and done at a single center, which may limit generalization to the other regions; however, findings are still significant and have reported moderate quality evidence. It suggests that there may be some likely to change evidence in future research. However, the evidence is somehow robust because general healthcare practice also guides professionals to educate their patients through informed decision-making to ensure evidence-based practice.

Weitzel (2020) highlighted that national regulatory policies are challenging and open access to the vaccination process in Chile because vaccinations are provided mostly in public vaccination centers [19]. It is not authorized to give vaccines indicated for travel-related problems such as rabies and polio. Similar issues have been highlighted and reported from Asian-origin countries [32]. The drawbacks of Weitzel's study (2020) are that it was single-centered and had one travel medicine specialist. The basic demographic features were comparable to those of travel medical centers in Europe and the United States [33-36].

Weitzel (2020) also examined the rise in rates of work-related consultations. It is common in places with restricted access to travel medicine. Weitzel indicates that a group of travelers and employers are more motivated to seek assistance in these restricted-access travel medicine places. These findings are also supported by Flaherty et al. (2017). They observed in a survey based on China reported unusually a smaller number of travel medicine practitioners. A team of researchers noticed more than 90% of work-related pre-travel health consultations in China despite a lower number of practitioners [37]. Moreover, Han et al. (2015) also found that there is a need to educate and have training programs for travel health professionals to enhance their knowledge. They must be qualified enough to guide travelers about how their pre-existing medical health conditions may affect their health during or after traveling. Therefore, there is a need for educational and training programs to ensure comprehensive pre-travel health consultation combined with individual-specific health strategies [23].

Walker et al. (2015) also addressed the issue of the limited time preparedness of business and occupational travelers to seek pre-travel health consultation. The authors suggested that there is a need to initiate earlier pre-travel health consultations (four to six weeks before) for these travelers who may have immediate traveling requirements and are short on time. There is a need to act proactively to prevent health risks due to prolonged stays [25]. Moreover, in another study by Rozenberg et al. (2019), research aimed to assess the function of mobile phone consultation in travel medicine and its impact on information memory, traveler happiness, and the amount of time allotted to each consultation. It is welcomed by travelers and patients, particularly those who are facing difficulties and challenges in a vacation to a low-income nation [20]. Similarly, Cowman (2012) also reported travel-related health consultations using mobile phones. However, they emphasized that online WhatsApp consultation should not be replaced by standard prolonged consultation. Online support should complement standard consultation to enhance the impact and scope of travel health consultation because satisfaction rates did not improve in the online support group [38]. Freedman et al. (2016) reported a similar issue that travel health consultation recollection of medical interactions is inadequate. Travelers frequently do not comprehend all of the issues raised. Therefore, standard consultation and online support should complement [39]. 

Tan et al. (2018) explained that it is challenging to determine the precise number of travelers for consultation as many of them may not experience illness or seek pre-travel health consultation. Therefore, the exact number of diarrhea patients is not known. Moreover, a large number of travelers did not get pre-travel health consultations [21]. Shaw et al. (2015) also found that 80% of travelers did not get post-exposure healthcare after returning from travel as compared to those who sought pre-travel consultations [24]. Similarly, Freedman et al. (2016) estimated that 20%-80% of passengers do not seek pre-travel consultation due to various reasons that are not known [39]. The strategies must be devised to know the underlying reasons to prevent the disease's development and progression, which may put other populations at risk.

Tan et al. (2018) also necessitate considering the following factors for pre-travel health consultation, including age, gender, race, residence, occupation, travel destination spot, unclean food, water, rural areas, hiking, and camping. Hill (2000) also examined the majority of patients who received treatment at travel health clinics seeking consultation for humanitarian service or parental job relocation. Business or corporate travelers who were on vacation from America were more likely to undergo examinations at a comprehensive medical facility before traveling to developing countries. They were provided appropriate consultation and guidance for the challenges and problems [40].

Vilkman et al. (2016) discovered that even with successful pre-travel preventive measures, a notable number of health problems were still seen when analyzing the immunization data. More precisely, an astonishing 76% of the participants in the study stated that they became sick on their travels, and 25% encountered ongoing symptoms and new issues within two days of coming back. The highest susceptibility to health issues was observed at the age of 31.5 years [22]. The percentage of participants in the research who experienced any symptoms while abroad (76%) was comparable to the prevalence among Americans (64%) [41] and the United Kingdom (64%) [42]. Although most of the disease symptoms originated abroad, the statistics revealed a surprisingly high number of new health concerns during the follow-up, with 32% of our patients reporting such issues. This percentage slightly surpasses the findings of previous literature that investigated the occurrence of the disease upon return and during follow-up. A strong correlation exists between young travelers who were experiencing health-related problems [43-45].

All of the studies have observational and retrospective designs except one, which is a randomized controlled trial [20]. One is multicentric, and other studies are single-centric, which may increase the ability to include diverse study representatives to generalize findings in the future through more multicentric studies with robust interventional-based study designs. The included studies were conducted by single travel medicine experts; most travel medicine consultations are not provided by a specific travel medicine specialist. Small sample sizes limit the statistical powers of studies. High dropout rates in follow-up questionnaires and missing data are also reported among studies, which limits the generalizability of findings. Chances of selection bias and recall bias are because some patients ask about travel illness after returning from the journey, which may increase the likelihood of bias. There is also a lack of selection of healthy traveling or non-traveling control groups to compare the results or findings. The possibility of data being missing or distorted is expected because of the usage of questionnaire-based data collection. The information on pre-travel health consultations is also limited because not all travelers seek pre-travel health consultations. The reviewer's ability to appropriately interpret the data was hindered by the insufficient information regarding the use of antibiotics for self-treatment of traveler's diarrhea and the lack of data on the number of diarrhea episodes averted through pre-travel health consultations.

Future recommendations

Future researchers are directed to conduct more RCTs to elevate the evidence strength. The RCTs should be focused on considering comparable healthy tourists and students as control groups to find the evidence authentically. Future researchers must focus on conducting controlled trials with a large sample size based on multi-centers, which will increase the validity and generalization of findings. Studies should be of diverse, inclusive, large sample size, which should be representative of the whole population to generalize the findings. Future researchers should focus on, with special consideration, consultants who are specialists in travel medicine rather than selecting general physicians in assessments of behavior or illness perception among travelers. The future studies direct focus on updating knowledge to incorporate guidelines to develop national policy-making to effectively prevent travelers from illness on foreign land. Further investigation of travelers’ perspectives on pre-travel health consultation and post-exposure management could provide insights on subsequent travel-related illnesses.

## Conclusions

While synthesizing evidence, it was found that the pre-travel health consultation is a multifaceted and essential approach. It also plays a vital role in preventing travel-related illness. Moreover, there are some predictors such as nationality, travel purpose, and time duration of stay while traveling; destinations are predisposing key factors that impact the pre-travel health consultation. The current approach is needed to be more specific and proactive to address health-specific risks experienced by travelers. However, early comprehensive consultations focusing on preventive measures, region-specific health risks, and timely immunizations are crucial to improving health outcomes. Moreover, enhanced guidance, awareness, and education of health professionals are also necessary to treat the complex medical needs of business and occupational travelers effectively. In addition to that, some challenges and limitations constrain pre-travel health consultations. For example, limited preventive vaccination facilitates, lack of national guidelines and practice of travel medicine, lack of travel medicine experts, convincing, campaigning, and providing awareness regarding pre-travel health consultation are also restricting its implementation. However, it is a comprehensive framework to enhance or improve outcomes related to the travel health of patients. Owing to limitations, future researchers are directed to conduct randomized controlled trials that strengthen the evidence by utilizing rigorous methodology, inclusive of large sample sizes, multicenter consideration to ensure generalization of findings, and tailoring effective strategies to improve overall well-being and health. 
